# Is There a Consensus on Air Travel Following Hip and Knee Arthroplasty?

**DOI:** 10.7759/cureus.43814

**Published:** 2023-08-20

**Authors:** Tobenna J Oputa, Amogh Patil, Joe B Amissah-Arthur, Joann Lum, Kathryn McLoughin, Qaisar Choudry, Ethan Caruana, Fayez Almari, Andrew Sloan

**Affiliations:** 1 Trauma and Orthopedics, Lancashire Teaching Hospitals NHS Foundation Trust, Preston, GBR; 2 Trauma and Orthopedics, Royal Blackburn Hospital - East Lancashire Hospitals NHS Trust, Blackburn, GBR

**Keywords:** flying, hip and knee replacement, air travel, deep vein thrombosis (dvt), total joint arthroplasty

## Abstract

Introduction

"When can I fly after my hip or knee replacement?" is a question frequently encountered by surgeons. Both air travel and arthroplasty increase the risk of venous thromboembolism (VTE); however, few studies examine the risk of air travel following arthroplasty. This study aimed to review the advice given to patients by surgeons, airlines, and insurance providers about flying after arthroplasty. We also review the current literature and available guidelines.

Materials and methods

A survey was sent to consultants with a special interest in hip or knee arthroplasty at 14 hospital trusts in the United Kingdom (UK) asking how long they would advise patients to avoid flying after surgery. We contacted all UK commercial airlines asking if they imposed any limitations on flying after arthroplasty. We contacted 15 UK insurance providers to determine whether they would provide insurance coverage following arthroplasty.

Results

A total of 110 knee surgeons and 105 hip surgeons were contacted. The response rate was 42% for hip surgeons and 44% for knee surgeons. Advised time to avoid flying varied widely from 14 to 180 days. A total of 22 airlines were contacted, and the response rate was 63% (n=14). Five airlines would not allow passengers to fly following arthroplasty and seven airlines required certification from a doctor. Fifteen insurance providers were contacted and the response rate was 73% (n=11). Seven insurance providers had restrictions on providing cover to passengers after arthroplasty.

Conclusion

Advice given to patients by surgeons, airlines, and insurance providers about flying following arthroplasty varies greatly. There is an absence of evidence-based guidelines to inform such advice. Further study is required to provide the evidence on which to base such advice. Therefore, we recommend that surgeons exercise caution when providing advice to patients.

## Introduction

“How soon can I fly after my hip or knee replacement?" this is a question frequently posed to hip and knee arthroplasty surgeons. Both flying and lower limb arthroplasty have been shown to increase a patient's risk of suffering a venous thromboembolic event. The risk of suffering from symptomatic venous thromboembolism (VTE) after prolonged air travel has been estimated to be around 1.9-5.2 per million person-days [1]. Similarly, it is estimated that around 3-4% of patients undergoing total hip arthroplasty will develop a symptomatic VTE within the first three months following surgery [2]. Patients with both of these risk factors are classed as being at an even greater risk [1-3].

Many arthroplasty surgeons advise patients to avoid flying for a brief period of time following surgery. However, there is an absence of any clear evidence in the literature to show that this is the case and this risk is in fact summative. Additionally, there are no strong evidence-based guidelines as to how long this time period should be. Patients are often advised to check with their airlines and travel insurance providers to see if they are permitted to fly and if their travel insurance is valid following surgery.

The aim of this study was to examine the advice given to patients undergoing hip or knee arthroplasty about air travel from surgeons, airline companies, and travel insurance providers. We also review the current guidelines and evidence base that may inform such advice, while considering the legal implications for surgeons.

## Materials and methods

All consultant orthopedic surgeons with a sub-specialist interest in hip or knee arthroplasty practicing at 14 hospital trusts in the United Kingdom (UK) were invited to complete an online survey. Consultants were asked how long they would advise patients, both with and without risk factors for VTE, to avoid taking long- and short-haul flights following both hip and knee arthroplasty. Long-haul flights were determined as any flight lasting longer than four hours. The 14 hospital trusts consisted of 21 hospitals, four of which were teaching hospitals, one was a national center for orthopedics and the remainder were district general hospitals. Among them, 7422 joint replacements were performed in the previous year (4068 hips and 3354 knees).

All UK commercial passenger airlines with type A licenses (airlines licensed to carry 20 or more passengers) were contacted by email or telephone. Airlines were asked if they had any restrictions on passengers flying following hip or knee arthroplasty and if passengers required any medical certification prior to flying.

We contacted 15 UK-based travel insurance providers by email or telephone. Insurance providers were asked if they had any restrictions on providing insurance cover to customers who had recently undergone hip or knee arthroplasty, and if an insurance claim would be valid should a customer traveling by air were to suffer from a VTE following hip or knee replacement surgery.

## Results

Surgeons

A total of 105 consultants with a sub-specialist interest in hip arthroplasty were invited to complete the survey. Forty-four surveys were completed (response rate: 42%). A total of 110 consultants with a sub-specialist interest in knee arthroplasty were invited to complete the survey. Forty-eight surveys were completed (response rate: 44%). Advice from surgeons varied widely both for patients with and without risk factors for VTE and for patients traveling on short and long-haul flights.

**Figure 1:**
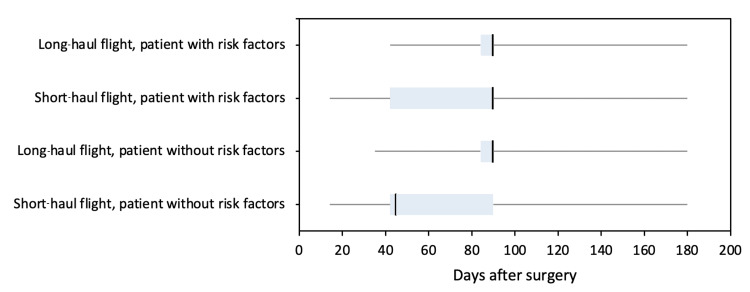
Boxplot analysis showing median, quartiles, and range of time advised to avoid surgery by consultants for short- and long-haul flights and patients with and without risk factors for VTE for both hip and knee arthroplasty. VTE: venous thromboembolism Time is given as number of days after surgery.

For patients with no risk factors for VTE, the advised time to avoid flying following both hip and knee arthroplasty ranged from 14 to 180 days for short-haul flights and 35 to 180 days for long-haul flights. The median time for short-haul flights was 45 days and 90 days for long-haul flights. For patients with one or more risk factors for VTE, advised time to avoid flying following both hip and knee arthroplasty ranged from 14 to 180 days for short-haul flights and 42 to 180 days for long-haul flights. The median time was 90 days for both long- and short-haul flights (Figure 1).

Airlines

A total of 22 airlines were contacted and 14 replies were received (response rate: 63%). Five (36%) of the airlines informed us that they had restrictions on passengers flying after hip or knee arthroplasty, while the other nine airlines had no restrictions. Three of these five airlines did not permit patients to fly for seven days following surgery. The fourth airline did not permit patients to fly for 10 days following surgery and the fifth required patients to wait 14 days after surgery before flying. Seven (50%) of the contacted airlines required medical certification for patients who wished to fly in a defined period after surgery. This was seven days for one airline, 10 days for three airlines, and 14 days for another airline. Also, two airlines did not specify what this time period was (Table 1).

**Table 1:** Number of airlines with time restrictions on flying after hip or knee arthroplasty or requiring patients to provide fit to fly certification.

Variables	Restriction on flying (number of airlines)	Certification required (number of airlines)
None	9	7
7 days	3	1
10 days	1	3
14 days	1	1
Unspecified	0	2

Insurance providers

A total of 15 insurance providers were contacted and 11 replies were received (response rate: 73%). Seven (64%) of these insurance providers informed us that they had restrictions on providing insurance coverage for patients traveling by air after hip or knee arthroplasty. Two of the insurance providers required certification from a doctor or surgeon that the patient was fit to fly. One company told us that coverage would be dependent on patient screening. The four remaining companies had time restrictions on when insurance coverage could be provided following surgery ranging from six weeks to two years (Table 2). When asked if a submitted claim would be valid for a patient suffering from VTE who has traveled by air following hip or knee arthroplasty, nine (82%) companies said they would accept the claim provided adequate information had been disclosed when cover was arranged.

**Table 2:** Restrictions on providing insurance coverage from various insurance providers.

Restrictions on providing insurance cover	Number of insurance providers
None	4
6-8 weeks	1
1 year	2
2 years	1
Once signed off by doctor/surgeon	2
Depends on screening	1

## Discussion

Findings

Our results demonstrate that there is a great disparity in the advice given to patients by surgeons about flying following hip or knee arthroplasty ranging from 14 to 180 days. There are also disparities between the rules and regulations of different airlines as to when patients are permitted to fly following hip or knee arthroplasty ranging from zero to 14 days. Similarly, there is a disparity among travel insurance providers as to whether insurance can be provided to customers who have recently undergone hip or knee arthroplasty ranging from zero days to two years.

Guidelines

Guidance from the National Institute of Health and Care Excellence (NICE) in the United Kingdom on deep vein thrombosis (DVT) prevention for travelers stratifies patients who have recently undergone major surgery into a group of patients at high risk. In such patients, NICE recommends that specialist advice should be sought and clinicians should recommend delaying or canceling trips. Where travel is unavoidable, patients should be given general advice on DVT prevention and advised to use graduated compression stockings or low molecular weight heparin when indicated [4].

NICE recommends that following hip or knee arthroplasty, patients should be advised to avoid long-haul flights for three months but that it may be possible to undertake short-haul flights after six weeks [4]. However, on review of the references of these guidelines, these recommendations only appear to be based on level five evidence [5]. One of their key outcomes is that there is a lack of evidence to support any recommendations made for air travel following arthroplasty [4].

The American Association of Hip and Knee Surgeons state that patients with a low risk of blood clots can travel soon after their joint replacement if appropriate measures are taken, such as DVT prophylaxis medication, compression stockings, and staying well hydrated. Higher-risk patients are advised to discuss their travel plans with their primary care doctor as well as their surgeon [6]. However, in the United Kingdom, several National Health Services provide leaflets to patients which recommend not flying within three months of surgery [7-9].

The Civil Aviation Authority (CAA) in the United Kingdom provides guidance to passengers on flying after different surgical procedures, but this does not extend to hip or knee arthroplasty [10]. There is; however, guidance provided for healthcare professionals on assessing fitness to fly following surgery. This recommends avoiding air travel for three months after both hip and knee arthroplasty [5].

Existing knowledge 

In a review of the literature, only two studies were identified reporting on the incidence of VTE in patients flying after hip or knee arthroplasty [11,12]. Neither study demonstrated patients to be at an increased risk. Ball et al., reviewed 608 patients on VTE chemoprophylaxis who traveled an average of 1377 miles at an average of 6.5 days postoperatively following hip arthroplasty (462 by airplane, 143 by car, and 3 by train) [11]. There were no reported deaths, no symptomatic pulmonary embolisms, and only five reported symptomatic DVTs (0.82%). Four of these five were in patients who had other significant risk factors for VTE. This study concluded that with chemical VTE prophylaxis, travel within six weeks of hip arthroplasty surgery is associated with a low rate of symptomatic DVT [11].

In a retrospective review of 1465 consecutive patients undergoing total hip and knee replacement surgery, Cooper et al. compared a cohort of 220 patients who traveled by air at a mean of 2.9 days postoperatively, with 1245 patients who did not travel by air postoperatively [12]. There was no statistical difference in the incidence of symptomatic deep vein thrombosis or pulmonary embolus or VTE overall between the two cohorts. This study concluded that allowing air travel after total joint arthroplasty appears to be a safe practice [12].

Implications

Our results have demonstrated that there is no clear consensus among orthopedic surgeons as to when patients should be allowed to fly following hip and knee arthroplasty. Advice offered by orthopedic surgeons varied widely and was neither standardized nor evidence-based. This is in part due to a lack of any relevant, consistent, and evidence-based guidelines to assist when making recommendations in such circumstances.

Courts in the United States and Australia have previously recognized an airline's liability for failing to adequately warn patients of the risk of suffering a DVT following international flights [13,14] and despite previous contradictory judgments, this remains a contentious issue in the United Kingdom [15]. Without a strong evidence base or consensus, many surgeons may be exposing themselves to litigation when giving such advice. In some scenarios, over half of the surgeons surveyed advised patients that they could fly sooner than was recommended by the guidelines from the CAA and NICE. We question how this may affect the defense of a surgeon in court if a patient developed a DVT after advising a patient that they could fly.

Furthermore, half the airlines surveyed and two of the 15 insurance companies required certification from a doctor before flying. Surgeons may be exposed to potential further litigation if a scenario occurs where a patient develops DVT after receiving documentation stating they were "fit to fly." In their advice to general practitioners about certifying patients as fit to fly, The Medical Protection Society recommends careful consideration of the wording of statements for airlines, and where possible recommends simply offering factual information only [16].

## Conclusions

We advise surgeons to be cautious when providing advice to patients about flying following hip and knee arthroplasty. We would recommend that it is stressed that there is currently no evidence as to when it is “safe” to fly, ensure that patients are aware of the potential risks, recommend necessary precautions, and also make this clear in any documentation provided for airlines and insurance companies. We would also echo advice from the Medical Protection Society that rather than certifying patients as “fit to fly” following surgery, surgeons should instead consider providing a factual letter outlining the above.

Due to the inherent difficulty and infeasibility of performing any randomized control trials, we recommend that a clinical consensus study is performed with the aim of producing guidelines to inform the advice given by surgeons.
